# Delivery strategies of cancer immunotherapy: recent advances and future perspectives

**DOI:** 10.1186/s13045-019-0817-3

**Published:** 2019-11-28

**Authors:** Zhongwei Zhao, Liyun Zheng, Weiqian Chen, Wei Weng, Jingjing Song, Jiansong Ji

**Affiliations:** 1 Key Laboratory of Imaging Diagnosis and Minimally Invasive Intervention Research, Affiliated Lishui Hospital of Zhejiang University/the Fifth Affiliated Hospital of Wenzhou Medical University /The Central Hospital of Zhejiang Lishui, Lishui, 323000 China; 2 Department of Radiology, Affiliated Lishui Hospital of Zhejiang University/the Fifth Affiliated Hospital of Wenzhou Medical University/The Central Hospital of Zhejiang Lishui, Lishui, 323000 China; 3 Department of Interventional Radiology, The Fifth Affiliated Hospital of Wenzhou Medical University, Affiliated Lishui Hospital of Zhejiang University, The Central Hospital of Zhejiang Lishui, Lishui, 323000 China

**Keywords:** Cancer, Immunotherapy, Delivery, Nanoparticle

## Abstract

Immunotherapy has become an emerging strategy for the treatment of cancer. Immunotherapeutic drugs have been increasing for clinical treatment. Despite significant advances in immunotherapy, the clinical application of immunotherapy for cancer patients has some challenges associated with safety and efficacy, including autoimmune reactions, cytokine release syndrome, and vascular leak syndrome. Novel strategies, particularly improved delivery strategies, including nanoparticles, scaffolds, and hydrogels, are able to effectively target tumors and/or immune cells of interest, increase the accumulation of immunotherapies within the lesion, and reduce off-target effects. Here, we briefly describe five major types of cancer immunotherapy, including their clinical status, strengths, and weaknesses. Then, we introduce novel delivery strategies, such as nanoparticle-based delivery of immunotherapy, implantable scaffolds, injectable biomaterials for immunotherapy, and matrix-binding molecular conjugates, which can improve the efficacy and safety of immunotherapies. Also, the limitations of novel delivery strategies and challenges of clinical translation are discussed.

## Introduction

**Fig. 1:**
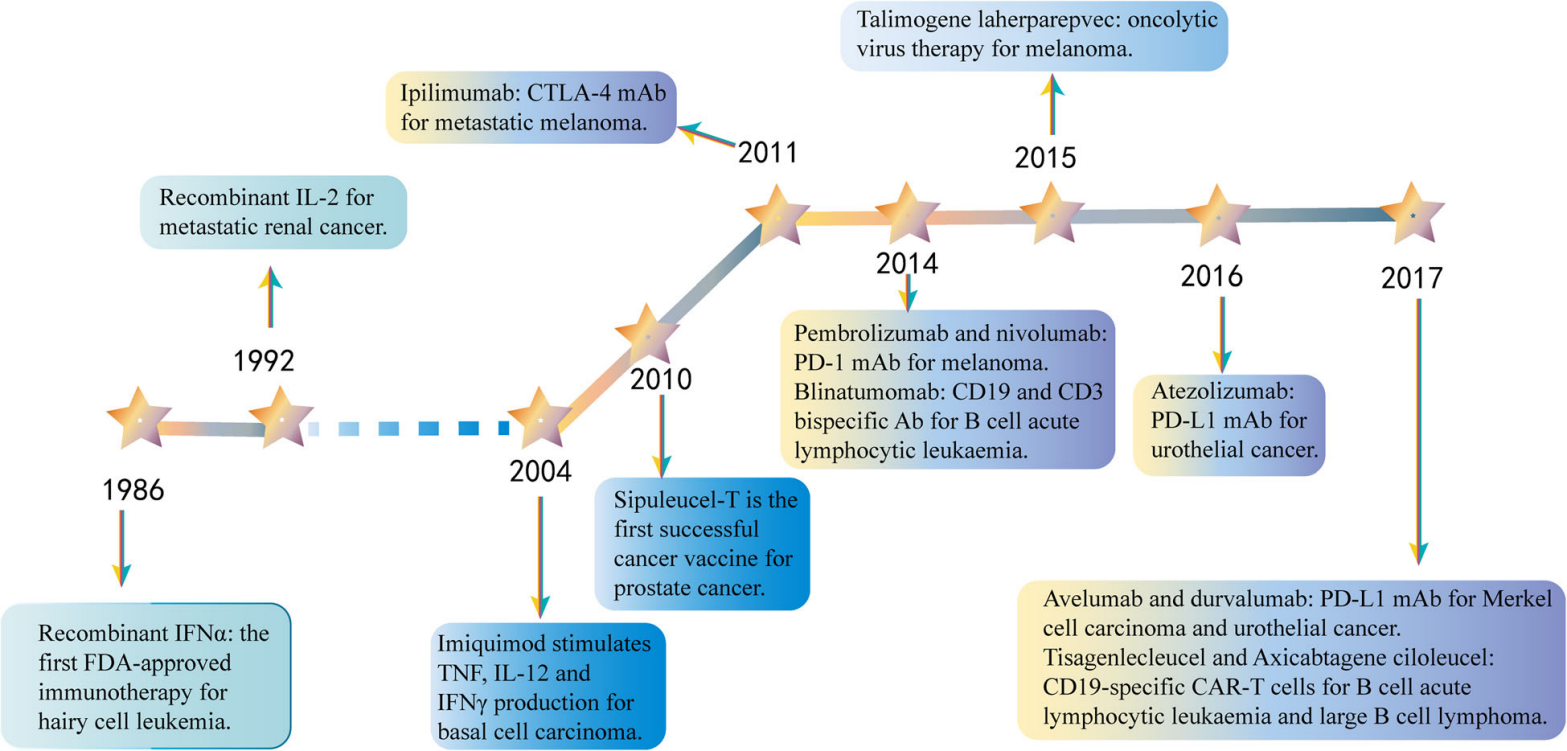
Timeline of FDA-approved cancer immunotherapies. *FDA* Food and Drug Administration, *IFN* interferon, *IL* interleukin, *mAb* monoclonal antibody, *CTLA*-*4* cytotoxic T lymphocyte antigen 4, *PD*-*1* programmed cell death 1, *PD*-*L1* PD-1 ligand 1, *CAR* chimeric antigen receptor

Cancer immunotherapy has revolutionized the treatment of cancer. Compared to chemotherapy and other drugs that directly kill tumor cells, cancer immunotherapy can stimulate and/or promote the immune system in the body to indirectly attack and kill tumor cells, with the goal of improving anti-tumor immunity while reducing off-target effects [1–3]. In 1986, the recombinant cytokine interferon-*α* (IFN*α*) was the first commercially available cancer immunotherapy approved by the US Food and Drug Administration (FDA) for hairy cell leukemia [4] (Fig. 1). Partial remission can be observed in some patients, but due to the short duration of treatment with IFN*α*, purine analogues quickly replaced IFN*α* and became the first-line treatment for hairy cell leukemia [5]. Subsequently, the FDA approved recombinant interleukin-2 (IL-2) for the treatment of metastatic renal cancer and metastatic melanoma in 1992 and 1998, respectively [1]. Although its application induces long-lasting complete responses in some patients, serious side effects, such as cytokine release syndrome (CRS) and vascular leak syndrome, come with high doses due to the short half-life of IL-2 [6–9]. As for the vaccines, sipuleucel-T, an autologous dendritic cell therapy, was the first successful therapeutic cancer vaccine approved in 2010 for prostate cancer [10]. However, its clinical translation was limited by some issues, including production complexity [11–14].

Strikingly, the monoclonal antibody (mAb) ipilimumab is a pioneering immune checkpoint inhibitor (ICI) targeting cytotoxic T lymphocyte antigen 4 (CTLA-4), which was approved in 2011 for metastatic melanoma [15]. Other immune checkpoint inhibitors, targeted programmed cell death 1 (PD-1) or its ligand, (PD-L1), and chimeric antigen receptor (CAR) T cell therapy have been created and used clinically [16–24]. The emergence of ipilimumab and CAR-T cell therapy is an epoch-making turning point in cancer immunotherapy, which is called a breakthrough in 2013 by Science [25]. Currently, a variety of immunotherapies have been approved for cancer treatment (Table 1). Therefore, as a promising therapeutic strategy, immunotherapy is considered to have the ability to treat or even cure certain cancer.

**Table 1:** Approved immunotherapies for cancer treatment

Class	Agent	Description	Indications
Cytokines	Intron A	Recombinant IFNα2b	Hairy cell leukemia, melanoma, follicular lymphoma, and AIDS-related Kaposi sarcoma
	Roferon-A	Recombinant IFNα2a	Hairy cell leukemia, chronic myelogenous leukemia, and AIDS-related Kaposi sarcoma
	Aldesleukin	Recombinant IL-2	Melanoma and kidney cancer
	Imiquimod	Stimulating TNF, IL-12, and IFNγ production	Basal cell carcinoma
Cancer vaccines	Sipuleucel-T	Autologous PBMCs activated with recombinant human PAP–GM-CSF	Prostate cancer
	Bacillus Calmette–Guérin	Strain of *Mycobacterium tuberculosis* variant bovis	Bladder cancer
Immune checkpoint inhibitors	Ipilimumab	CTLA-4 mAb	Melanoma
	Pembrolizumab	PD-1 mAb	Melanoma, non-small-cell lung cancer, Hodgkin lymphoma, advanced gastric cancer, microsatellite instability-high cancer, head and neck cancer, and advanced urothelial bladder cancer
	Nivolumab		Melanoma, bladder cancer, classical Hodgkin lymphoma, colorectal cancer, hepatocellular cancer, non-small-cell lung cancer, kidney cancer, squamous cell carcinoma of the head and neck, and urothelial cancer
	Atezolizumab	PD-L1 mAb	Urothelial cancer and non-small-cell lung cancer
	Avelumab		Merkel cell carcinoma and urothelial cancer
	Durvalumab		Urothelial cancer and non-small-cell lung cancer
CAR-T cells	Tisagenlecleucel	CD19-specific CAR-T cells	B cell acute lymphocytic leukemia and non-Hodgkin lymphoma
	Axicabtagene ciloleucel		Large B cell lymphoma

Although immunotherapy has made significant advances, the clinical applications of immunotherapy encounter several challenges associated with safety and efficacy. For example, in terms of safety, immunotherapy can cause fatal adverse effects in some patients, including autoimmune reactions, CRS, and vascular leak syndrome [26, 27]. Regarding the efficacy, only a minority of patients respond to immunotherapy [28, 29]. In addition, major immunotherapies were initially evaluated in hematological malignancies because solid tumors faced delivery barriers such as complex tumor microenvironments. Given this, a few of immunotherapies, such as activated cytokines and ICIs, have been granted by the FDA for the treatment of solid tumors [30]. Interestingly, the FDA has not yet approved CAR-T cell therapy for solid tumors, but researchers are actively developing CAR-T cells that are highly specific for solid tumor [31, 32].

Novel strategies, especially improved delivery strategies, are able to more effectively target tumors and/or immune cells of interest, increase the enrichment of immunotherapies within the lesion, and reduce off-target effects. Some materials, such as lipids, polymers, and metals, have been used to exploit delivery strategies [33–36]. At present, new delivery strategies are being researched and developed for immunotherapy, including nanoparticles, scaffolds, and hydrogels [37]. These delivery platforms offer many advantages for immunotherapy compared to separate therapeutic agents. On the one hand, the delivery systems can be designed to achieve spatiotemporal control of the treatment and to protect the therapeutic cargo until it is delivered and accumulated within the target cells [38, 39]. On the other hand, delivery platforms, for instance implants, have been utilized to achieve localized delivery of therapeutic drugs in a controlled manner, and cell therapy has been used to minimize toxicity related to systemic administration [40–42].

Here, we briefly describe five major types of cancer immunotherapy, including their clinical status, strengths, and weaknesses. Then, we introduce novel delivery strategies that can improve the efficacy and safety of immunotherapies. Also, the limitations of novel delivery strategies and challenges of clinical translation are discussed.

## Cancer immunotherapy: classification, clinical status, advantages, and disadvantages

### Cytokines: interferons, interleukins, and GM-CSF

Interferons, interleukins, and granulocyte-macrophage colony-stimulating factor (GM-CSF) are the three major cytokines applied in immunotherapy [26]. The cytokine recombinant IFN*α* was approved for clinical use in 1986, marking the cytokine as a pioneer in immunotherapy [4]. Unlike immune checkpoint inhibitors, cytokines directly boost the activity and growth of immune cells.

In response to microbial pathogen infections, interferons are generally produced by immune cells and thereby induce the maturation of various immune cells, such as macrophages, dendritic cells (DCs), natural killer (NK) cells, and lymphocytes, to exert immune responses [43–46]. Angiogenesis in the extracellular tumor space can also be suppressed by interferon-activated immune cells [44, 47]. Moreover, interleukins stimulate the activity and growth of T cells [23, 48–50]. GM-CSF utilizes two mechanisms to achieve the goal of enhancing immune responses. One is to promote T cell homeostasis, thereby enhancing T cell survival, and the other is to support dendritic cell differentiation, which in turn allows these cells to express tumor-specific antigens [51]. In addition to the three major cytokines mentioned above, the researchers are also studying related agonists, which activate immune cells through intracellular mechanisms. For instance, agonists of toll-like receptors 7/8 (TLR7/TLR8) stimulate antigen-presenting cells (APCs) to improve anti-tumor immunity, while stimulator of interferon genes (STING) agonists are utilized to trigger pro-inflammatory cytokine production and other type I interferon immune responses [52, 53].

However, due to the short half-life of cytokines, treatment often requires high-dose bolus injections, which can lead to serious side effects, including CRS and vascular leak syndrome [26]. In addition, cytokine therapy can lead to autoimmune attacks against healthy tissues by inducing the death of activated T cells and facilitating the survival of regulatory T cells [27]. Currently, increasing research is attempting combination therapies, including the combination of two or more cytokines, the combination of cytokines with immune checkpoint inhibitors or chemotherapies, with the goal of reducing the side effects of high therapeutic doses required for individual treatment [44].

### Cancer vaccines: nucleic acids, dendritic cells, and neoantigens

Nucleic acid therapy has become a promising cancer vaccine, including DNA-based or RNA-based vaccines. The vaccine depends on exogenous nucleic acids being transported into the target cells [54, 55]. Mechanistically, APCs usually take up DNA or mRNA and translate them into antigens, which are presented to T cells to stimulate their activation. Activated T cells then attack tumor cells expressing antigens of interest [54, 55]. Moreover, the mRNA vaccines encode pro-inflammatory cytokines (e.g., IL-12) or trafficking-related molecules to regulate DC functions [56–58]. A significant increase in DC immunostimulatory activity can be achieved by using mRNA vaccines encoding costimulatory molecules (e.g., CD83) [59, 60]. Intratumoral administration of TriMix mRNA vaccines, which do not encode tumor-associated antigens, activate CD8α^+^ DCs and tumor-specific T cells, thereby slowing tumor growth in mouse models [61]. Continued antigen availability during vaccination promotes both high antibody titers and germinal center (GC) B cells and T follicular helper (TFH) cell responses [62]. This process may be a contributing factor to the efficacy of the nucleoside-modified mRNA-LNP vaccines [63, 64]. Due to the difficulty of nuclear delivery and immunogenicity, DNA vaccines have failed in many clinical trials [65, 66]. Instead, the mRNA vaccines induce protein expression without crossing the nuclear barrier. Also, mRNA is non-infectious and unintegrated into the genome [54, 67]. Currently, non-replicating and self-amplifying mRNAs are two types of mRNA vaccines in which non-replicating mRNAs are used more frequently [54, 68, 69]. However, mRNA is easily degraded due to the universality of RNase. To increase mRNA stability, several sequence modifications have been applied, including poly(A) tail additions, the use of 5′ caps, the incorporation of pseudouridine sequences, and optimized 5′ and 3′ untranslated regions (UTRs) [70–72]. In addition, transfection agents or delivery platforms are needed to mediate intracellular delivery and protect it from degradation [54, 73]. Collectively, improvements in delivery technologies can greatly enhance the efficacy and safety of nucleic acid vaccines, such as increased intracellular (mRNA) and intranuclear (DNA) delivery.

Dendritic cell vaccines are the most studied type of cell-based cancer vaccine [74]. They are derived from patients’ dendritic cells that are modified to express tumor-associated antigens and directly stimulate T cells to target cancer cells [74]. Due to its ability to prolong overall survival, sipuleucel-T, a dendritic cell vaccine, was approved for the treatment of prostate cancer in 2010 [10]. However, other dendritic cell-based vaccines are frustrating in clinical trials. Despite high safety, they lack efficacy [75]. Therefore, in order to achieve the purpose of improving efficacy, on the one hand, dendritic cells expressing high levels of targeted antigens can be identified, and on the other hand, delivery to relevant lymph nodes can be enhanced [74, 76].

The neoantigens are tumor-specific antigens that are only present in cancer cells. Cancer vaccines based on neoantigens can increase the number of neoantigen-specific T cells in vivo to enhance adoptive anti-tumor immunity. Currently, neoantigen-based vaccines are being studied as novel cancer immunotherapies because they can enhance the immune responses to tumor cells [77, 78]. Preclinical studies have shown that the neoantigen-based cancer vaccines are effective and feasible in mouse tumor models, including melanoma, colon cancer, and glioma [68, 79–82]. For example, neopeptides containing IDH1 (R132H) p123-142 mutation region were synthesized and bound to transgenic human MHC-II molecules. The results from IDH1 (R132H) mutant glioma mouse model showed that the neopeptide vaccine could trigger rapid and effective mutation-specific anti-tumor immune responses [82]. Also, clinical trials of neoantigen-based vaccines are ongoing for various tumors [83–87]. In six melanoma patients, a synthetic long peptide (SLP) vaccine against up to 20 individual neoantigens was used. Results showed that four patients had no tumor recurrence within 25 months after vaccination, and two patients with relapse obtained tumor regression after receiving PD-1 antibody [85]. In addition, neoantigen-based vaccines also show the potential therapeutic effects in human glioblastoma [86, 87]. Keskin et al. found that the number of neoantigen-specific CD4^+^ and CD8^+^ TILs were increased in eight glioblastoma patients vaccinated with multi-epitope neoantigen vaccine in a phase I clinical trial [87] Meanwhile, personalized neoepitope vaccine (APVAC 2) mainly caused CD4^+^ Th1 cell responses in 15 patients with glioblastoma [86]. Therefore, neoantigen-based vaccines have a promising future in cancer immunotherapy.

### Agonists targeting T cell surface receptors

Co-stimulatory receptors (i.e., CD28) and tumor necrosis factor receptor (TNFR) family members, including TNF receptor superfamily member 9 (i.e., 4-1BB), TNF receptor superfamily member 4 (i.e., OX40), and glucocorticoid-induced TNFR-associated protein (GITR), are the most commonly targeted T cell surface receptors [88]. As for co-stimulatory receptors, agonistic antibodies bind to these co-stimulatory receptors and thereby induce T cell growth and exert tumoricidal activity [27]. For members of the TNFR family, agonistic antibodies may play a role through the NF-*κ*B, JNK, and PI3K-AKT pathways [89]. Therefore, agonists can specifically bind to surface receptors of T cells and activate intracellular signaling pathways, thereby promoting T cell proliferation, survival, and exerting effector functions of killing tumor cells [90].

Currently, some clinical trials have used agonistic antibodies to target different receptors [89]. Ongoing phase II trials include agonistic antibodies targeting 4-1BB (e.g., utomilumab and urelumab) and antibodies targeting OX40 (PF-04518600, BMS-986178, and INCAGN-01949, etc.) [91–93]. However, dose-limiting toxicity also occurs on agonistic antibodies because agonists can trigger the activity of unwanted immune cell subtypes to attack healthy cells [88]. Based on this, researchers are evaluating the toxicity related to specific doses and dosing schedules, and are developing delivery technologies to solve this issue. For instance, in mouse models, anti-4-1BB antibodies immobilized to liposomal nanoparticles showed lower toxicity and increased intratumoral accumulation compared to freely delivered antibodies [94]. Therefore, advanced delivery technology should be developed for agonistic antibodies in the future. This technology is capable of both controlling the duration of exposure and simultaneously inducing multivalent T cell activation.

### Immune checkpoint inhibitors: mAbs targeting PD-1/PD-L1 and CTLA-4

To date, immune checkpoint inhibitors (ICIs) have been the most studied class of cancer immunotherapies, including PD-1/PD-L1 blockade and CTLA-4 blockade [3, 19]. Normally, immune checkpoints act as an immune brake to keep appropriate immune responses and simultaneously keep healthy tissues away from immune attack [95]. CTLA-4, as a co-inhibitory molecule, regulates the degree of T cell activation. Once CTLA-4 binds to its ligand (CD80 and CD86), it impairs T cell function and thus contributes to tumor progression. Blockade of CTLA-4 can repair T cell function and enable T cells to exert tumor-killing ability [96]. In addition, upon inflammation, T cells are activated and express PD-1, allowing them to recognize abnormal cells [97]. In the tumor microenvironment (TME), PD-L1 expressed by tumor cells binds to PD-1 on T cells to inactivate T cells, thereby allowing tumor cells to escape T cell recognition and clearance [18]. Thus, mAbs targeting PD-1 or PD-L1 can disrupt this interaction and improve T cell anti-cancer immunity [98].

Currently, one CTLA-4 inhibitor and five PD-1 or PD-L1 inhibitors have been approved by the FDA for the treatment of various cancers [19]. Compared to conventional chemotherapies, overall survival rates have indeed improved [99]. However, the disadvantages still exist. Firstly, serious adverse effects can occur in many organs due to systemic administration of ICIs [100–102]. Secondly, only a small percentage of patients respond to ICIs, and many patients do not respond. Low responses may be associated with low numbers of tumor infiltrating T cells and adaptive resistance to ICIs [103, 104]. Finally, different TMEs have various mechanisms of immunosuppression [105].

### CAR-T cell therapy

In recent years, CAR-T cell therapy has achieved remarkable success in clinical use and has received much attention. CAR-T cells are derived from T cells of the patient’s blood, which are modified in vitro to express specific CARs that recognize tumor cell antigens and are re-transferred to the same patient. After injection, tumor cells are specifically recognized and killed by CAR-T cells [106, 107]. CAR-T cells can maintain their activity for more than a decade after injection and are typical of onetime therapy compared to other therapies [108, 109]. The original target for CAR-T cells is CD19, as this molecule is often expressed on B cell leukemias and lymphomas and is only expressed in immature B cells. Therefore, “on-target, off-tumor” activity can cause B cell aplasia, which can be alleviated by immunoglobulin replacement therapy [110].

At present, two CD19-targeted CAR-T cell therapies are FDA-approved for clinical use: tissuelecleucel for acute lymphocytic leukemia and diffuse large B cell lymphoma and axicabtagene ciloleucel for diffuse large B cell lymphoma [111, 112]. The clinical success of CD19-targeted CAR-T cell therapy has motivated researchers to design CAR-T cells for different antigens or a combination of several antigens in order to facilitate their widespread use [106, 113, 114]. However, there are some challenges in the wide application of CAR-T cells. First, the production of CAR-T cells is time consuming, expensive, and technically challenging [115]. Second, CAR-T cells can result in severe side effects such as cytokine release syndrome and neurotoxicity [116, 117]. Moreover, in solid tumors, except for glioblastomas that express EGFRvIII, these engineered cells are less effective and do not persist [118–120]. Therefore, combinational therapies and novel delivery strategies are required to increase their applicability to solid tumors.

## Novel delivery strategies of immunotherapy with improved efficacy and safety

### Nanoparticle-based delivery of immunotherapy

**Fig. 2:**
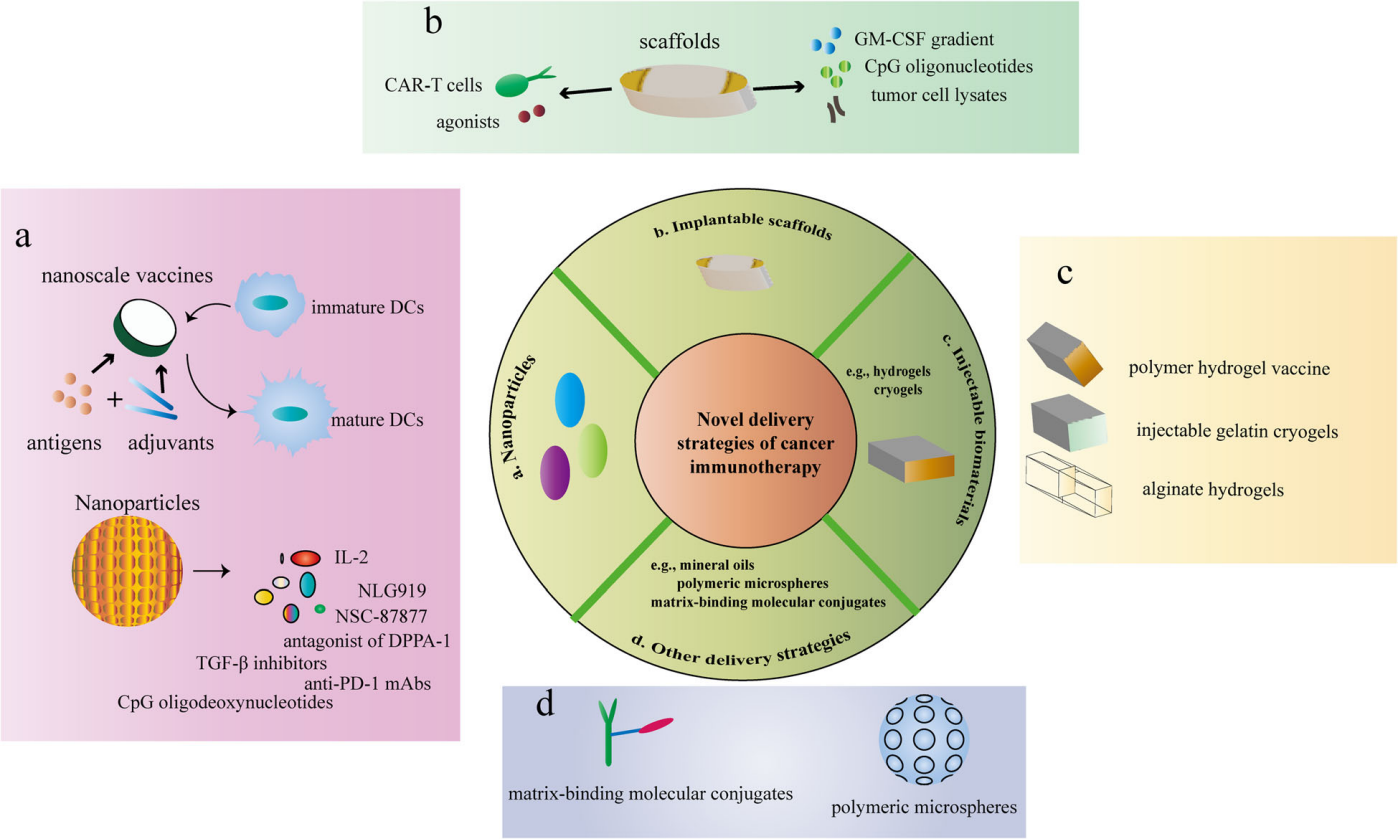
Novel delivery strategies of immunotherapy with improved efficacy and safety. **a** Nanoparticle-based delivery of immunotherapy. Nanoparticles can mediate the delivery of vaccines. The most researched nanoscale vaccines were antigen (e.g., proteins and peptides)-TLR agonist fusion vaccines. Amphiphilic nanoscale vaccines have also been created which are composed of antigen or adjuvant cargo attached to the tail of the lipophilic albumin. High-density lipoprotein mimic nanodiscs conjugated to neoantigen peptides and adjuvants were developed. Nanodisc-based vaccines can greatly increase the efficiency of co-delivery of antigens and adjuvants to lymphoid tissues and thus induce DCs maturation. Moreover, nanoparticle-mediated delivery targets multiple inhibitory signals in the tumor microenvironment. Therapeutic peptide assembly nanoparticles, an antagonist of d-peptide programmed cell death ligand 1 (DPPA-1), were fabricated and co-assembled with NLG919 (an inhibitor of indoleamine 2,3-dioxygenase 1 (IDO-1)). In addition, nanoscale liposome polymer gels (nLGs), including TGF-β inhibitors and IL-2, were designed. And nano-cocoons can control the release of anti-PD-1 antibodies and CpG oligodeoxynucleotides, which can prevent cancer recurrence and prolong mouse survival. NSC-87877, a potent Shp1 and shp2 protein tyrosine phosphatases inhibitor, was packaged in the nanoparticles. Nanoparticles carrying NSC-87877 were conjugated to the surface of tumor-specific T cells and stimulated T cell expansion. **b** Implantable scaffolds for the delivery of immunotherapy. Implantable scaffolds are biomaterials that can be preloaded with a variety of chemical reagents, biological factors, or cells. The scaffolds are typically implanted through a small surgical procedure into the subcutaneous or resected sites. The bioactive agents can be controlled to release in the implanted scaffold, and the immune cells are typically recruited to access the scaffolds for further bio-programming. For example, poly (lactide-co-glycolide) (PLG) polymer scaffolds were designed to contain GM-CSF, CpG oligonucleotides, and tumor cell lysates as recruitment factors, risk signals, and antigen sources, respectively. Alginate scaffolds can co-deliver CAR-T cells with cyclic dinucleotide (CDN) STING agonists to treat solid tumors. **c** Injectable biomaterials for immunotherapy. Injectable biomaterials include hydrogels and cryogels. The advantage of these materials is that they can be positioned anywhere the needle can reach without the need for surgical implantation. This is a relatively simple and minimally invasive procedure that does not require much technical expertise and avoids unnecessary tissue damage and a series of complications related with inflammatory wound response. **d**. Other delivery strategies: matrix-binding molecular conjugates, mineral oils, and polymeric microspheres. Matrix-binding molecular conjugates have been developed to accumulate within and around tumors, reducing systemic drug exposures and side effects. For example, with a water-soluble amine-sulfhydryl crosslinker, checkpoint inhibitors bound to a peptide from placental growth factor 2 (PLGF2), which has a particularly high affinity for a variety of matrix proteins. These conjugates were more localized in the extracellular matrix around the tumor tissue, leading to delayed tumor growth and extended survival. Mineral oils and polymeric microspheres are designed for local and controlled release. A commercially available light mineral oil blend, Montanide ISA 51, has been applied in clinical trials for immunotherapy. This mixture was utilized to prepare sustained release formulations that delivered agonistic anti-CD40 antibodies locally. In addition, biodegradable polymer microparticle formulations have also been developed to deliver immunomodulatory antibodies locally and continuously, including PLHMGA

Nanoparticles can mediate the delivery of vaccines (Fig. 2 a). The most researched nanoscale vaccines were antigen (e.g., proteins and peptides)-TLR agonist fusion vaccines [121, 122]. The combination of TLR agonists and antigen allows the antigen and adjuvant to be co-delivered to the same immune cell. A representative study attached TLR7/8 agonists to polymer scaffolds and demonstrated that the polymer-TLR7/8 agonists with low agonist density could self-assemble into particles ranging in diameter from 10 to 20 nm. The production of cytokines in the lymph nodes was higher than that of unformulated TLR7/8 agonists [123]. Amphiphilic nanoscale vaccines have also been created which are composed of antigen or adjuvant cargo attached to the tail of the lipophilic albumin [124]. The use of these nano-vaccines in vivo can significantly accumulate in lymph nodes and reduce systemic distribution. The results showed that T cell activation was increased by 30-fold, anti-tumor immunity was greatly enhanced, and systemic toxicity was greatly decreased. This delivery strategy is simple and widely used to increase the efficacy and safety of the vaccine at the same time. In addition, high-density lipoprotein mimic nanodiscs conjugated to neoantigen peptides and adjuvants were developed [81]. Nanodisc-based vaccines can greatly increase the efficiency of co-delivery of antigens and adjuvants to lymphoid tissues and thus maintain antigen presentation to DCs. Compared to soluble vaccines, nanodiscs frequently induce neoantigen-specific immune responses at frequencies up to 40-fold. In animal tumor models, nanodiscs cleared tumors when combined with anti-PD-1 and anti-CTLA-4 therapies [81]. Therefore, nanodisc-based vaccine is promising in personalized cancer immunotherapy.

Nanoparticle-mediated delivery targets multiple inhibitory signals in the tumor microenvironment (Fig. 2 a). A nanoparticle-based strategy was developed to suppress both the immune checkpoints and the tryptophan metabolism. Therapeutic peptide assembly nanoparticles, an antagonist of D-peptide programmed cell death ligand 1 (DPPA-1), were fabricated and co-assembled with NLG919 (an inhibitor of indoleamine 2,3-dioxygenase 1 (IDO-1)) [125]. The nanoparticles exhibited a spherical shape as well as sustained release of the drug, which was promoted in the presence of acidic pH and enzymes. In the tumor stroma, the nanoparticles swelled and subsequently collapsed, and DPPA-1 and NLG919 were locally released, which is beneficial to the activation and survival of cytotoxic T lymphocytes (CTLs). Treatment with dual immune checkpoint inhibitors increased the percentage of CD8^+^ T cells in the tumor and in turn exerted potent anti-tumor immunity, inhibiting the growth of melanoma. In summary, this study demonstrates that nanoparticles provide new opportunities for cancer immunotherapy by targeting multiple inhibitory signals of the tumor microenvironment.

In addition, nanoscale liposome polymer gels (nLGs), including TGF-β inhibitors and IL-2, were designed [126]. Notably, nLGs continuously released IL-2 and TGF-β inhibitors into the tumor microenvironment, improved the activity of NK cells and CD8^+^ T cells, and thereby enhanced anti-tumor immune responses. The results indicated that tumor growth was slowed and the survival rate of tumor-bearing mice was increased. Therefore, the efficacy of nLGs in cancer immunotherapy is closely related to the activation of innate and adaptive immune responses. Moreover, nano-cocoons can control the release of anti-PD-1 antibodies and CpG oligodeoxynucleotides, which can prevent cancer recurrence and prolong mouse survival [127]. Another strategy of triggering T cells by covalently coupling nanoparticles to free sulfhydryl groups on T cell membrane proteins has been reported to efficiently deliver compounds into T cell synapses [128, 129]. Shp1 and shp2 protein tyrosine phosphatases downregulate TCR activation in synapses. NSC-87877, a potent inhibitor, was packaged in the nanoparticles. Nanoparticles carrying NSC-87877 were conjugated to the surface of tumor-specific T cells and stimulated T cell expansion. Therefore, this study offers a novel strategy to suppress the immune pathway that impairs T cell activation.

Also, a dual pH-responsive multifunctional nanoparticle system was created to combine immunotherapy and chemotherapy [130]. R848, a synthetic analogue regulating Toll-like receptor, was loaded into the poly(l-histidine) core, while doxorubicin (Dox) bond to the shell of hyaluronic acid through acid-decomposable hydrazine bonds. Ionization of poly (l-histidine) near pH 6.5 and breakage of hydrazine bond at pH 5.5 promoted the release of R848 and Dox in the tumor microenvironment. R848-encapsulated nanoparticles have strong immunoregulatory activities against DCs. Therefore, the synergistic administration of drugs and adjuvants can enhance the effect of immunotherapy and chemotherapy for breast cancer.

### Implantable scaffolds for immunotherapy

Implantable scaffolds are biomaterials that can be preloaded with a variety of chemical reagents, biological factors, or cells. The scaffolds are typically implanted through a small surgical procedure into the subcutaneous or resected sites. The size of the implants is consistent with a small tablet or pill. The bioactive agents can be controlled to release in the implanted scaffold, and the immune cells are typically recruited to access the scaffolds for further bio-programming [131, 132] (Fig. 2 b).

Poly (lactide-co-glycolide) (PLG) polymer scaffolds were designed to contain GM-CSF, CpG oligonucleotides, and tumor cell lysates as recruitment factors, risk signals, and antigen sources, respectively. Specific dendritic cell populations can be recruited and programmed [133]. The implanted scaffold must be maintained in the body for more than 7 days, with the aim of triggering adequate immune responses and thus inhibiting tumor growth. In brain tumor models, it has been shown that anti-tumor efficacy is closely related to the ability of the implant to contact the tumor tissue and build a GM-CSF gradient [134, 135]. PLG scaffolds are constantly being improved in design and application to deliver a variety of agonists. And scaffolds in combination with ICIs can enhance CTLs activity [98, 99]. Currently, a vaccine called WDVAX (ClinicalTrials.gov identifier: NCT01753089) is undergoing phase I clinical trial evaluation in patients with stage IV melanoma [136]. It can be expected that specific antigens or synthetic neoantigens can be developed to achieve personalized vaccines [137].

Recent studies have shown that alginate scaffolds can co-deliver CAR-T cells with cyclic dinucleotide (CDN) STING agonists to treat solid tumors [138]. In the mouse pancreatic tumor model, due to the limitations of CAR-T cell monotherapy, intravenous injection of CAR-T cells alone failed to eliminate the tumor. However, when alginate implants are combined with CDN, the therapeutic efficacy of CAR-T cells can be obviously improved [139]. It is worth noting that the implants, loaded with CAR-T cells without CDN, more than doubled the survival rate of mice compared to CAR-T cell therapy alone. However, scaffolds were not able to completely eliminate the tumor, indicating the need to use STING agonists in order to promote long-lasting anti-tumor immunity [138]. Implanted scaffolds co-released CAR-T cells and STING agonists, which are able to clear tumors with an average survival increase of 37 days. Interestingly, tumor re-challenge in tumor-clearing mice indicated that they had established complete immunity in their bodies, with no pancreatic tumor regrowth.

Additionally, scaffold-based cancer vaccine delivery is a new strategy for cancer immunotherapy [140]. Porous 3D scaffolds were prepared by cross-linking collagen and hyaluronic acid. It can deliver both gemcitabine and cancer vaccines [141]. The inhibition of tumor immunosuppression induced by myeloid-derived suppressor cells is mediated by gemcitabine. The recruitment and activation of dendritic cells, the increase in the number of CD4^+^ and CD8^+^ T cells, and the enhancement in IFN-γ production are all attributed to cancer vaccines. Systematic anti-tumor immunity was produced in the model of primary breast cancer after operation, which prevented in situ recurrence and lung metastasis. Therefore, compared with bolus vaccine formulations, scaffolds exhibit better systemic anti-tumor immunity and tumor growth inhibition in delivering vaccines, adjuvants, or other drugs.

### Injectable biomaterials for immunotherapy

Injectable biomaterials include hydrogels and cryogels [142, 143]. The advantage of these materials is that they can be positioned anywhere the needle can reach without the need for surgical implantation. This is a relatively simple and minimally invasive procedure that does not require much technical expertise and avoids unnecessary tissue damage and a series of complications related with inflammatory wound response [144] (Fig. 2c).

An injectable polymer hydrogel vaccine was created as an immune initiation center, and hydrogels were also loaded with chemoattractants and immunomodulators to improve DCs infiltration and immune reprogramming [145, 146]. This injectable therapy improved two-fold survival in B cell lymphoma models [146]. Subsequently, a two-layer hydrogel/microsphere complex was developed for delivering exogenous immune cells [147]. An injectable alginate-based system established a hydrogel in situ that was capable of carrying exogenous DCs [148]. The ability to deliver immunostimulatory molecules via bulk encapsulation from a self-gelling system was also explored. In recent years, injectable gelatin cryogels from natural collagen facilitated the infiltration and expansion of immune cells and controlled the release of GM-CSF [149]. Moreover, the alginate hydrogel system was utilized to form larger pores relative to the more standard nanoporous alginate systems [150]. These macroporous alginate hydrogels greatly increased cell infiltration, and when containing GM-CSF, the injected hydrogels recruited a population of millions of immature DCs [150]. Subsequent studies have shown that directly conjugated peptide antigens can be delivered by the same pore-forming alginate hydrogels preloaded with GM-CSF, leading to the recruitment and reprogramming of antigen-specific T cells [151].

An alginate hydrogel combination therapy was reported for local delivery of celecoxib and anti-PD-1 mAbs into tumors [152]. Utilizing the anti-inflammatory properties and intrinsic anti-tumor activity of celecoxib, the efficacy of anti-PD-1 mAbs can be improved by counteracting the harmful anti-PD-1-induced chronic inflammation [153]. It was demonstrated in the melanoma models that celecoxib or anti-PD-1 mAbs was delivered separately from subcutaneously injected alginate hydrogels, which obviously inhibited tumor growth compared with drug injection alone [152]. This indicated that the hydrogels sustained higher local drug concentration and continued to deliver. In addition, the simultaneous delivery of celecoxib and anti-PD-1 mAbs significantly enhanced anti-tumor efficacy, as manifested by significantly reduced tumor size, as well as complete regression of some mouse tumors [152]. Also, compared to local or systemic administration of free gemcitabine and anti-PD-L1 antibodies, local injection of hydrogel reduced postoperative tumor recurrence and prolonged survival in a melanoma mouse model [154]. Additionally, the combination of DC vaccines and anti-PD-1 mAbs is also delivered by peptide hydrogel [155].

Therefore, injectable biomaterials are a complement to implantable scaffolds, and both delivery strategies have shown impressive therapeutic results.

### Other delivery strategies: matrix-binding molecular conjugates, mineral oils, and polymeric microspheres

Matrix-binding molecular conjugates have been developed to accumulate within and around tumors, reducing systemic drug exposures and side effects (Fig. 2d). For example, with a water-soluble amine-sulfhydryl crosslinker, checkpoint inhibitors bound to a peptide from placental growth factor 2 (PLGF2), which has a particularly high affinity for a variety of matrix proteins [156]. In the murine models with melanoma and breast cancer, these conjugates were more localized in the extracellular matrix around the tumor tissue compared with the unmodified inhibitors after peritumoral administration, which led to delayed tumor growth and extended survival [156]. In addition, these conjugates boosted systemic anti-tumor immunity and decreased side effects related to systemic administration of ICIs. Also, the matrix-binding molecular conjugate is scalable to enable local delivery of ICIs to other tumor sites of the body that are difficult to be reached by systemic administration.

A commercially available light mineral oil blend, Montanide ISA 51, has been applied in clinical trials for immunotherapy [157]. This mixture was utilized to prepare sustained release formulations that delivered agonistic anti-CD40 antibodies locally [158]. In a mouse model of lymphoma, local injection of the formulations eliminated both local and secondary tumors [158]. This method requires only a lower dose of antibody to stimulate T cells and thereby avoid systemic toxicity. In addition, due to local lesions caused by Montanide ISA 51 at the injection site of mice, including inflammation, swelling, and granuloma, biodegradable polymer microparticle formulations have also been developed to deliver immunomodulatory antibodies locally and continuously [159, 160]. For example, poly(d,l-lactic-co-hydroxymethyl glycolic acid) (PLHMGA), a biodegradable polymer, was used in a mouse colon cancer model for slow and sustained release of anti-CD40 and anti-CTLA4 antibodies [159]. It is worth noting that local injection of PLHMGA microparticles can control the release of antibodies for more than 30 days and has considerable efficacy [159]. These polymeric microspheres are characterized by complete reabsorption in vivo with lower serum antibody levels, which provides a durable immunotherapy delivery system while reducing the risk of systemic side effects [159].

## Limitations of novel delivery strategies for immunotherapy

Although novel delivery strategies hold potential for cancer immunotherapy, some limitations still remained that need to be further considered. Firstly, the size of the nanoparticles influences their biodistribution and pharmacokinetics in vivo. Nanoparticles, less than 200 nm in size, can proceed with more freedom in the lymphatic circulation to deliver antigens and/or adjuvants, thus increasing the likelihood of activating APCs. Secondly, the toxicity characteristics of nanoparticle-based immunotherapy require adequate attention. It is unclear whether nanoparticles increase immune activation while also increasing autoimmune responses. Once nanoparticles can induce more autoimmune side effects, methods are needed to minimize the side effects. Since nanoparticles can better activate dendritic cells and T cells via co-stimulating multiple signaling pathways, the translation of nanoparticle-based delivery for immunotherapy requires an accurate assessment of their toxicity. Moreover, nanotechnology can increase the complexity and cost of manufacture and commercialization, which is detrimental to the clinical translation of nanoparticle-based immunotherapy.

In addition, confirmation of biocompatibility and degradation of biomaterials, such as scaffolds and hydrogels, is important. As noted above, scaffolds and hydrogels are used locally and systemic toxicity may be limited. However, due to the biological material itself, an acute inflammatory reaction may still be triggered. Of course, chronic inflammatory reactions may emerge due to the continuous degradation of biological materials.

As for the implantable scaffolds, there are also some disadvantages. The scaffolds are rigid and brittle, prone to breakage, and require surgery to implant into the subcutaneous areas. Prefabricated alginate scaffolds, although resorbable without brittle problems, still require invasive surgical procedures to implant tumor resection sites. Thus, the implantable scaffolds are limited to the accessible location of the surgical procedure and is not easily implanted anywhere it is desired. And they usually have to be maintained at their implant sites for a sufficient period of time to function. However, their persistence may potentially impair normal organ function. For example, compared to controls without scaffolds, alginate implants have some damage to pancreatic activity for treating pancreatic tumors [138]. Moreover, injectable materials have the disadvantage that the selected biological material must have the mechanical property to form a liquid or gel with the aim of passing through the needle, severely limiting the type of materials.

## Challenges of clinical translation and future directions

### Selection of animal models

The selection of animal models is crucial. Many cancer immunotherapy regimens have proven effective in animal models, but rarely enter clinical trials. Therefore, there is an urgent need for a humanized in vivo model to ensure that the most promising candidates enter clinical trials and are still satisfactory. Subcutaneous tumor-bearing models, patient-derived xenograft (PDX) models, and genetically engineered mouse (GEM) models are three common animal models for studying human disease [161]. Each mouse model has its own key strengths and weaknesses. Subcutaneous implantation of cell lines is relatively simple, but does not replicate human disease well. The PDX models need immunocompromised animals, and it is therefore challenging to convert the results of immunotherapy into a person with a complete immune system. In addition, in the GEM models, immunocompetent mice are designed to develop diseases spontaneously, best replicating human disease and evaluating immunotherapy. However, designing and controlling experiments can be challenging due to the spontaneity of disease formation. Thus, a perfect in vivo model can reflect the natural state of cancer and precisely analyze preventive or therapeutic interventions to demonstrate true efficacy and safety.

### Design guidelines, including material selection and cost and complexity of production

Biomanufacturing is the foundation for the development of cancer immunotherapy delivery strategies and requires greater resource acquisition and cost reduction. Producing large-scale industrial samples at a cost that is affordable to patients is a challenge, especially in the early stages. Therefore, several design guidelines, including treatment stability, scalability, and cost and complexity of production, are fundamental issues to consider for clinical translation [162]. The selection of materials is also related to the process of clinical translation. Compared with unapproved materials, the use of FDA-approved materials for delivery may be faster to enter the clinic. This is beneficial for lipid- and polymer-based materials because the FDA has approved several materials as drug delivery platforms [163, 164]. For example, ongoing melanoma clinical trials utilize and evaluate FDA-approved lipids for delivering mRNA to dendritic cells (NCT02410733). However, the challenge is that the FDA has not yet approved mRNA-based agents. Therefore, the application of the therapy to the clinic may take longer. Moreover, ongoing clinical trials are also evaluating an injectable scaffold (WDVAX) for delivery of cancer vaccines (NCT01753089).

### Future directions

There are two aspects that can be further improved in the future. One is to study novel delivery strategies to expand and engineer the ex vivo cell therapy. Another is that biological materials should be created to increase the ex vivo expansion of T cells [165–167]. For example, microfluidics-based technology can accelerate the intracellular delivery of macromolecules to the ex vivo immune cells [168, 169]. The technique is very efficient in providing nucleic acids and macromolecules to immune cells (T cells, B cells, DCs, and macrophages), at speeds of up to about 1 million cells per second. The principle is that when cells pass through a point of contraction within a microfluidic channel, these cells undergo rapid mechanical deformation that instantaneously destroys the membrane of the immune cell, thereby absorbing macromolecules in the buffer [170]. Furthermore, in order to generate APC mimic scaffolds for T cell expansion, mesoporous silica microrods are coated with a fluid lipid bilayer, anti-CD3 and anti-CD28 antibodies, and IL-2 [171]. By replicating how APCs present these signals in vivo, these scaffolds greatly facilitate polyclonal amplification of primary human and mouse T cells. Similar in vivo efficacy can be found in mouse models with lymphoma [171]. The use of biological materials to improve the expansion and function of T cells can reduce off-target effects by increasing migration to target tissues in future studies, thereby improving T cell delivery.

## Conclusions

Cancer immunotherapy has become an emerging way of cancer treatment. Cancer immunotherapy as a whole is rapidly developing. However, the delivery technology for cancer immunotherapy is still in its infancy. Novel delivery strategies that improve immunotherapy are introduced for controlled release, local delivery, and increased stability. Many of the delivery technologies described not only provide a way for improving immunotherapy but also provide a way to overcome the inherent heterogeneity of cancer. We can envision that these technologies will be increasingly recognized in the future. For instance, many delivery systems, such as nanoparticles, scaffolds, mesoporous silica, and hydrogels, can be utilized to accommodate a variety of therapeutic agents that are selected on the basis of patient-specific targets. This personalized treatment will offer the potential of curing cancer patients. Therefore, continuous advancement in drug delivery will contribute to the wider application of cancer immunotherapy in the foreseeable future.

## Data Availability

Not applicable.

## Abbreviations

APCs: Antigen-presenting cells
CAR: Chimeric antigen receptor
CRS: Cytokine release syndrome
CTLA-4: Cytotoxic T lymphocyte antigen 4
DCs: Dendritic cells
FDA: Food and Drug Administration
GEM: Genetically engineered mouse
GM-CSF: Granulocyte-macrophage colony-stimulating factor
ICI: Immune checkpoint inhibitor
IDO-1: Indoleamine 2,3-dioxygenase 1
IFNα: Interferon-α
IL-2: Interleukin-2
mAb: Monoclonal antibody
NK: Natural killer
PD-1: Programmed cell death 1
PDX: Patient-derived xenograft
PLGF2: Placental growth factor 2
STING: Stimulator of interferon genes
TFH: T follicular helper
TLR7/TLR8: toll-like receptors 7/8
TME: Tumor microenvironment
TNFR: Tumor necrosis factor receptor
UTRs: Untranslated regions
